# Physician attitude toward depression care interventions: Implications for implementation of quality improvement initiatives

**DOI:** 10.1186/1748-5908-3-40

**Published:** 2008-09-30

**Authors:** Rachel Mosher Henke, Ann F Chou, Johann C Chanin, Amanda B Zides, Sarah Hudson Scholle

**Affiliations:** 1 Department of Health Care Policy, Harvard Medical School, Boston, MA, USA; 2 Thomson Reuters, Cambridge, MA, USA; 3 College of Public Health, University of Oklahoma, Oklahoma City, OK, USA; 4 College of Medicine, University of Oklahoma, Oklahoma City, OK, USA; 5 National Committee for Quality Assurance, Washington, DC, USA; 6 Revolution Health, Washington, DC, USA

## Abstract

**Background:**

Few individuals with depression treated in the primary care setting receive care consistent with clinical treatment guidelines. Interventions based on the chronic care model (CCM) have been promoted to address barriers and improve the quality of care. A current understanding of barriers to depression care and an awareness of whether physicians believe interventions effectively address those barriers is needed to enhance the success of future implementation.

**Methods:**

We conducted semi-structured interviews with 23 primary care physicians across the US regarding their experience treating patients with depression, barriers to care, and commonly promoted CCM-based interventions. Themes were identified from interview transcripts using a grounded theory approach.

**Results:**

Six barriers emerged from the interviews: difficulty diagnosing depression, patient resistance, fragmented mental health system, insurance coverage, lack of expertise, and competing demands and other responsibilities as a primary care provider. A number of interventions were seen as helpful in addressing these barriers – including care managers, mental health integration, and education – while others received mixed reviews. Mental health consultation models received the least endorsement. Two systems-related barriers, the fragmented mental health system and insurance coverage limitations, appeared incompletely addressed by the interventions.

**Conclusion:**

CCM-based interventions, which include care managers, mental health integration, and patient education, are most likely to be implemented successfully because they effectively address several important barriers to care and are endorsed by physicians. Practices considering the adoption of interventions that received less support should educate physicians about the benefit of the interventions and attend to physician concerns prior to implementation. A focus on interventions that address systems-related barriers is needed to overcome all barriers to care.

## Background

Major depressive disorder is a leading cause of disability in the US [1]. Nearly one in eight individuals has an episode of depression once in their lifetime, and 18.8 million adults suffer from a depressive illness each year [2]. Most individuals with depression seek treatment in the primary care setting [3,4]. Despite effective treatments that can be delivered in this setting, less than half of individuals with depression are appropriately diagnosed, and few of those receive adequate treatment [5]. Inadequate care has been attributed to barriers encountered by primary care physicians when treating the disorder [6]. The social and economic burden of depression [7] makes improving the quality of depression care in the primary care setting an important goal.

Interventions based on the chronic care model (CCM), a comprehensive framework for health services for the chronically ill, have been promoted to address barriers and improve the quality of depression care [8-13]. There are six elements of the CCM: delivery system design, decision support, clinical information systems, self management support, health systems, and community resources. Depression care interventions based on the CCM commonly promoted in research and demonstration projects include enhancing patient education, facilitating structured diagnostic instruments and screeners to identify cases and monitor symptoms, integrating mental health specialists into primary care, implementing care managers, and providing primary care access to mental health specialty consultation [14-16].

While the CCM has proven effective in improving the quality of chronic care treatment in research settings and effectiveness trials [17], we know little about whether physicians believe depression interventions address barriers to care. Ultimately, physician use and endorsement of these interventions will determine whether they are implemented successfully and sustained over time.

Our study examines primary care physicians' views on obstacles to providing depression care and CCM-based interventions promoted to address those barriers. We investigate two questions. First, which barriers are the most problematic from the physicians' perspective in today's practice? Previous empirical studies identifying barriers were conducted in the 1990s [18-22], and new or different barriers may have emerged since that time. Second, do physicians use and endorse the interventions? Inadequate physician buy-in is likely to jeopardize successful implementation. We address these questions using a qualitative analysis of interviews with primary care physicians about depression care.

## Methods

### Data

Data were collected as part of a study funded by The Robert Wood Johnson Foundation (RWJF) Depression in Primary Care: Linking Clinical and System Strategies program. This project sought to test the feasibility of implementing a primary care depression performance-based reward program. The study protocol was approved by the Chesapeake Institutional Review Board, and participating physicians gave informed consent.

### Sample and Recruitment

We recruited physicians using two methods to maximize the geographic and practice diversity of participants as well as the diversity in exposure and training in quality improvement interventions. The study group included both physicians who were high and low performers in depression care. First, we recruited physicians from health care organizations in the Southeast, the Western, and the Mid-Atlantic regions participating in performance measurement programs. Organizations with greater exposure to performance measurement programs were more likely to have practice tools available for caring for chronically ill patients. We identified a clinical champion at each practice who helped recruit individual physicians within each practice. Each site chose to use a different recruitment method. One site recruited physicians by verbal contact, another site sent an email to all physicians who were using an electronic medical record system compatible with a care management database, while the last site identified and sent a recruitment letter to physicians that had a higher percentage of patients with a depression diagnosis. Physicians in these three organizations were interviewed at their clinical care site. Second, we recruited physicians from a national database of physicians who had recently achieved or applied for recognition for diabetes care by the American Diabetes Association/National Committee for Quality Assurance Diabetes Physician Recognition Program (DPRP). Depression is a common co-morbidity of other chronic illnesses such as diabetes and heart disease, and physicians who have success in managing patients with diabetes were likely to have experience with models of care for chronically ill patients but were not necessarily high performers in depression care. These physicians worked in large and small practices that ranged from rural to urban settings throughout the U.S. and were interviewed by telephone.

### Interview

We developed an interview guide to assess barriers and facilitators to providing depression care according to evidence-based guidelines. We included prompts to assess use and attitude toward commonly promoted interventions based on the CCM: enhanced patient education, structured diagnostic instruments, depression screeners, integrated mental health specialists, care managers, and mental health specialty consultation. A panel of mental health experts reviewed and critiqued the guide, and we then piloted it by interviewing a primary care physician who was not included in our study cohort to ensure clarity of question phrasing. We made minor modifications to the guide after this interview as well as after interviewing physicians from the first site to eliminate unproductive questions and add questions that facilitated discussion of important constructs.

Two study team members conducted the one-hour semi-structured interviews with physician participants between January and April 2006. One team member took the lead in asking questions and following-up on responses while the other took notes and made sure that all of the important constructs were discussed. We allowed participant's responses guide the discussion instead of adhering to strict interview guide sequencing. All interviews were audio-recorded and transcribed with the consent of the participants.

### Analysis

Our analysis followed a grounded theory approach, a systematic process that enables researchers to identify broad concepts from the data [23]. Two members of the research team independently reviewed notes and transcripts from the interviews as they were completed to identify key concepts and themes related to understanding barriers and facilitators to depression care. The team members met to develop a preliminary coding scheme, resolving any inconsistencies through discussion. All transcripts were then coded using this scheme by one team member with Atlas software v5 [24]. The coder identified quotations based on her understanding of the dialogue rather than coding pre-determined chunks of the transcripts. The length and span of each quotation varied accordingly.

To enhance reliability, a second team member independently coded a subset of the transcripts manually, also identifying quotations based on meaning. This team member highlighted quotations and assigned codes in the margins. The two team members met to compare results. When the team members identified unique quotations or differed on choice of code, they discussed inconsistencies and reached consensus on which coding scheme to use.

The coding scheme was modified throughout the coding process to include additional concepts not captured by preliminary codes. Emergent themes were identified from retrieval of coded data and discussed during team meetings. Blocks of coded data were summarized in the results.

## Results

### Participants

The final sample consisted of 24 primary care physicians. Forty-two percent of the physicians were female and all were family practitioners or internists (Table 1). Of the 17 recruited from three health care organizations from the Southeast, West, and Mid-Atlantic regions, ages ranged from 33 to 55 years with a mean of 44 years. Twenty-four percent practiced in multi-specialty clinics, and 69% had five or more physicians in their group. Slightly different demographic information was collected on the DPRP physicians because they did not participate in the data abstraction component of the study. Mean years in practice for the DPRP physicians was 17.3 years, and all DPRP participants were male.

**Table 1:** Physician and practice characteristics

**Characteristic**	**Distribution**
Physicians	
Age*	44 y (range 33–55)
Years in practice**	17.3 y
Sex	58% male, 42% female
Specialty	71% internal medicine, 29% family/general practice
	
Physicians" practice environment*	
Practice specialty	6% solo practice, 71% single specialty group, 24% multi specialty group
On-site mental health availability	59% on-site mental health, 6% access at another site within medical group, 35% no on-site mental health
Medical record type	18% paper record only, 35% paper record supplemented with electronic data, 35% electronic medical record handles all functions, 12% electronic medical record with separate ordering or data system

*Age and physicians" practice environment describes all physician participants except DPRP physicians. The data were not collected for DPRP participants because they were not required to abstract data for the first part of the study.

** Years in practice describes physician participants from the DPRP sample.

Although all physicians were aware of depression care interventions discussed in the interviews, physicians had varied experiences with them. Several physicians worked in practices currently or previously involved in formal quality improvement programs related to use of the CCM. A few practices had selected intervention components such as on-site mental health specialists and/or access to care managers. Several practices facilitated the use of the Patient Health Questionnaire (*e.g*., PHQ-9) depression scale [25]. Other practice differences included the use of electronic medical records and the availability of staff trained to educate patients.

### Barriers to care

Our qualitative analysis identified six barriers to depression care:

1. Diagnosing depression is more difficult than diagnosing other conditions.

'There might be times when depressed symptoms are a normal response to circumstances and not clinical depression. It would be nice if we could somehow distinguish those.'

Many physicians reported that diagnosing depression is difficult because there is no standard lab test to quantify severity and patients may not express their symptoms in a clear or predictable way. Physicians expressed concern that patients may underreport symptoms from fear of stigma or exaggerate symptoms to gain attention. Further, some noted that determining whether a patient suffered from an adjustment reaction, bipolar disorder, or symptoms secondary to a medical illness required further evaluation for which they did not have the time or the expertise.

2. Patient resistance and poor compliance.

'As a physician, I only have so much control. I can do my best. I could do all of this! I can ask about substance abuse; I can give them the medication; I can schedule them a three-week follow-up. But if they don't show up I can't go to their home and get them.'

Nearly all physicians reported patient resistance to depression diagnosis and treatment. Physicians noted that the stigma associated with antidepressant use was not as prevalent as it once was, while stigma associated with psychotherapy continued to be a major barrier to referral. Patient failure to continue antidepressant treatment after experiencing symptom relief against recommendations was a source of physician frustration. Physicians also mentioned that low energy levels and motivation associated with depressive episodes led to poor adherence.

3. The mental health system is fragmented and difficult to access.

'There have been several times that I really thought somebody needed to see a psychiatrist and not a counselor, and I picked up the phone with the patient in my office to call the 1–800 number. I tell them I am the physician and I want them to see psychiatry, and they say, "Number one, we can't talk to you, and number two, they have to go to a counselor first." So, the only way that someone can see a psychiatrist is to go through whatever mental health number that the patient has to call and they have to see a counselor first, and then only that person can then send them to psychiatry. So, it's a little bit difficult. You never know when this happens.'

Physicians indicated that managing patients with mental illness was different from caring for those with a physical illness because of the organization of the mental health care system. Noted differences in access to mental health care compared to physical health care included a more burdensome administrative process, a longer waiting period for an appointment, reduced availability of specialists (particularly psychiatrists), and inadequate coordination between physicians and mental health specialists. One obstacle experienced by a few physicians was inability to make mental health specialty appointments on behalf of their patients because of insurance policies. Physicians noted that assisting with referrals was sometimes crucial for patients with depression due to reduced functioning associated with this disorder, yet they had fewer referral options than for those with other conditions.

4. Insurance coverage for depression treatment.

'If you have a Medicaid patient that comes in with an emotional issue you have to tell them, "Sorry, I can't see you for this."'

Insurance coverage was often insufficient to meet the needs of patients with depression. Physicians said that mental health visit limits and high out-of-pocket costs for mental health specialist visits hindered referrals. Physicians also said that treatment options were extremely limited for patients with no or inadequate drug coverage.

Medicaid and Medicare coverage for mental illnesses was described as problematic. Physicians said patients with Medicaid had limited access to mental health specialists because insufficient reimbursement reduced the number of practitioners willing to accept Medicaid, and public mental health clinics often had long waiting lists even for patients with urgent needs. Some physicians were unable to treat patients with Medicaid themselves due to practice and state policies. Physicians noted that patients with Medicare often were deterred by high coinsurance rates for mental health visits. Physicians also expressed concern about reimbursement downgrades from Medicare when billing for depression.

5. Lack of mental health expertise.

'I have a patient that can't get the [psychiatric] care they need and I'm just not sure what to do next. I find it frustrating and I almost don't want to see them any more because, I'm embarrassed to say, I just don't know what to do.'

Physicians felt pressured to provide care for patients with depression despite being stretched beyond their expertise and available resources. Reasons accounting for this barrier include patient preference for seeing a primary care physician rather than a specialist because of the time and financial burden of specialty care, stigma concerns, and greater comfort in seeing a provider with whom they already had a relationship. They also said that lack of specialist availability sometimes prevented them from referring patients who would benefit from it. Consequently, physicians noted patients with severe and complex mental illnesses often were left in their care despite their lack of expertise.

6. Competing demands and other responsibilities as primary care provider.

'If you took a diabetic, heart disease patient with cholesterol, and you did what's recommended for screening, it would take seven hours. So, it's really difficult in primary care to juggle it all and, unfortunately, a person's diabetes and chest pain take precedence in an internal medicine practice.'

Physicians described multiple responsibilities during office visits. When depression is first diagnosed, it is often within the context of a visit for another problem. Further, patients diagnosed with depression may come in for follow-up visits for other chronic conditions or for acute problems in addition to depression. While physicians expressed comfort discussing a series of physical conditions in quick succession, they noted difficulty handling depression this way because of its sensitive and emotional nature. Further, physicians reported patients with co-morbid conditions may require additional motivation and encouragement to ensure therapeutic adherence for both depression and other conditions.

'There's only a certain amount of time and energy that one can devote toward a visit. And anytime you add something, you're probably going to have to subtract something else.'

Beyond troublesome multi-issue visits, physicians were overwhelmed with their responsibilities as primary care physicians in general. They felt they lacked the time to screen for depression and manage the care according to depression guidelines. In particular, they felt they only had enough time to schedule frequent follow-up visits with the most severely depressed patients. Making phone calls as a substitute for in-person visits was not considered a viable alternative because they were not paid for telephone calls and were concerned about the time required to reach patients consistently.

### Physician use and attitude toward interventions

In this section, we describe physician attitudes about the usefulness of each intervention.

### Depression screening

Experts recommend the use of formal depression screening tools in coordination with effective follow-up and treatment to increase accurate identification of depressed patients [26]. Screening tools, such as the two-question Patient Health Questionnaire (PHQ-2), can be brief, easy to use, and administered by practice staff. Physicians can then follow up with a complete assessment of patients who screen positive for depression.

Some physicians advocated screening by staff before seeing the physician as a way to make patient visits more efficient. They reported if a patient screened positive, they could address depression with the patient at the beginning of the visit. Physicians felt this was important because depression was often the key to the patient's overall health and should be addressed in conjunction with treatment plans/goals associated with other conditions. Discussing depression first also eliminated the possibility that the patient would bring it up at the end of the visit, thereby prolonging the visit.

One physician who worked in a practice with a co-located mental health specialist noted that the presence of this individual made 'screening easier' because patients who screened positive would be seen by someone on site (the co-located mental health specialist) and managed appropriately.

Physicians were comfortable screening new Medicare patients for depression as mandated by the Centers for Medicare and Medicaid Services in the initial preventive physical exam questionnaire. However, few physicians endorsed screening all patients for depression because screening was time consuming and may encounter resistance from patients who already must complete multiple pre-exam forms. There was also concern that screening all patients may prompt discussions unrelated to the purpose of the visit among patients who were not depressed.

'You're going to get a lot of people who will [screen positive] and that's really not what they're here for. And some people like to have illness. I mean, I don't know how to explain that, but I think a lot of people will put a half a check, or "sometimes," and then it's hard to ignore it. And you just get everybody that way. And maybe in the real world, that's OK, but I think that's just going to open a Pandora's box, and you're going to get everybody who's ever felt depressed, or everybody who isn't sleeping, and every day would be like that.'

Instead of screening everyone, some supported targeting screening to at-risk populations, such as those with chronic conditions.

### Structured assessments

Experts recommend that physicians use structured assessments such as the PHQ-9 to facilitate diagnosing depression [25,27]. Use of the assessments has been found to yield a detection rate almost double that of routine physician diagnosis of depression [28]. The PHQ-9 can also be used to monitor patient response to treatment because it is a valid measure of treatment outcomes [28].

'Where I personally find standardized questionnaires helpful is when the person comes in and says, "Oh, I'm just fatigued. Things just don't go right. There must be something wrong with my thyroid." I think then getting them a standardized questionnaire is helpful, because I can say, "look at your answer and look at your score. This really is in the depressed range."'

All physicians were familiar with structured assessments, but few used them routinely to detect and monitor depression. More often, physicians used these tools selectively with patients for whom they thought they would be helpful. Notably, all providers who worked in the organization where the use of PHQ-9 was encouraged and easily integrated into the electronic medical record were routine users of the PHQ-9. Routine users reported many benefits including improved communication with patients about depression, enhanced patient involvement in symptom monitoring, simplified diagnostic process, and reduced burden of convincing patients of their depression diagnosis (patients perceived the severity score calculated from responses to structured assessments as more objective than clinical judgment). Routine users did not find the assessments cumbersome or time-consuming to use.

'If it's somebody you've known for ten years, and they've got disturbance in sleep and appetite and they're not enjoying their usual activities, and they are getting worse and worse for more than six weeks, eight weeks, you know this person is probably developing major depression and so you don't need to say, let me leave the room so you can fill out this survey, you just don't do that – if you went to a doctor that you've known for ten years and they use that, you would probably be like, "No, I'm telling you, I'm depressed"'

Many physicians who did not routinely use structured assessments wanted more information before incorporating them. Others chose not to use assessment tools or used them selectively because they remained unconvinced of their usefulness. Physicians described a tension between using assessment tools and asking questions for diagnosis. Some felt patients preferred not to respond to structured inventories because they viewed them as impersonal and thus may not be truthful or clear in their response to items. Others suggested that using structured assessments was redundant and inefficient because they typically probe for cardinal depression symptoms during patient interviews with open-ended questions and 'small talk'[29].

'Now, I do use a formalized screening tool when I'm seeing possible bipolar. It's normally helpful with that. But, as far as depression, I see a lot of it and it's fairly easy to detect with just nine basic questions.'

### Patient education

Practice guidelines recommend educating patients to overcome patient resistance to screening and treatment and to ensure treatment adherence [30]. Providing information about the cause, symptoms, and natural history of depression, treatment options including risks and benefits, anticipated outcomes, potential difficulties with compliance, and early warning signs of relapse is likely to improve adherence and outcomes [31,32].

Most physicians reported educating patients to some degree about the prevalence and biological basis of depression to reduce treatment resistance. One physician said telling patients about a billboard quote 'Depression is a chemical – it is not part of your character' attenuated stigma concerns. Other physicians noted that using structured depression assessments were useful in educating patients about the symptoms, engaging patients in treatment, and demonstrating progress. Except for physicians who had access to care managers, education appeared brief and oriented toward convincing the patient of their diagnosis and describing treatments because of time constraints. Further, education appeared limited to the first visit because physicians said they had limited time to provide extensive follow-up visits. Physicians with care managers relied on them to provide most or all of the education after making a diagnosis.

'Depression requires a lot of time. The [counselor] gets one hour with patients each time and can talk about different social things that could help with the depression and can go over other behavioral modifications and so forth, and I barely have time to get the diagnosis and to write a script.'

### Mental health integration

Experts recommend that primary care practices integrate on-site mental health specialists to enhance coordination of care [13]. Physicians who had access to on-site mental health professionals relied extensively on them, reporting greater information sharing, increased referral completion rate, and promotion of comprehensive and consistent treatment. Problems with co-located mental health specialists included long wait for appointments, availability limited to one or two days a week, and non-acceptance of Medicaid.

'Before [the co-located mental health specialist] came, it was very challenging to call the outpatient psychiatrists to get appointments, and then once the patient goes there, it mainly seems like they are seen by the residents, and then each time they go, there's a different doctor, so, patients don't like that, and ended up coming back here. So, I think we are very blessed and lucky [to have the co-located mental health specialist].'

All physicians who did not have access to on-site professionals indicated a need for it. To cope with not having on-site professionals, some physicians created their own 'integrated system' by making personal connections with therapists to help matching patients to available therapists when issuing referrals. Physicians also used their connections with mental health specialists to reduce delays in obtaining care for their patients. Some physicians tried to improve referral completion by activating the patient and telling the patient what to expect from a therapy visits to reduce apprehension.

Primary care physicians said they rarely received reports from specialists after patient referral. Some depended on patients to tell them if their mental health specialist had recommended a new or changed medication prescription. Those who did not feel comfortable collecting this information from patients had to make special efforts to contact specialists directly with variable success.

### Mental health consults

Facilitating primary care physician communication with psychiatrists for advice with complex cases has the potential to increase physician comfort in providing evidence-based depression care. Only a minority of primary care physicians in our sample had access to psychiatrists for advice; some through a telephone hotline set up by the insurance company, others through 'curbside consultations', informal meetings with co-located mental health specialists [33]. Of those with access, few utilized this resource. Those who contacted psychiatrists for consult indicated that it allowed them to become more educated and comfortable delivering depression treatment.

'If we have somebody that we're really concerned about, and don't know what to do, we can page him. He'll call us right back, help us to know what to do with that patient right then, and then pick up the pieces later.'

Of those without access, none expressed interest in it as a way to reduce the effect of barriers. Resistance to increasing access can be illustrated by one physician's report that when her practice implemented a telephone consultation system, few used it, despite initial concern about high volume of calls. While the reasons for physician lack of interest in obtaining consultations were unclear, time constraints or perceived inefficiency may be one factor.

'It's complicated [to use consults] because the patient you are going to run into trouble with is somebody that needs two or three drugs or that has failed things in the past. It's never just, "They didn't do well on this. Can we do this?" It's always this mish-mash of things. [Consults] may be helpful occasionally, but in general, they are almost going to have to see the person and hear the whole long Gone with the Wind story.'

### Care management

Current literature has stressed the benefits of non-physician follow-up monitoring to facilitate care coordination, symptom monitoring, and informational support to ensure compliance and efficacy in achieving self-care [10]. Care managers, whose role is coordinating disease management for patients, can improve depression outcomes by increasing rates of treatment, speeding response to treatment failure, and reducing the likelihood of patients' prematurely discontinuing care.

'We used to have a care manager – we could send her a note and she would call them at one week, and three weeks, and four weeks and get feedback if they're having side effects. That was excellent because she'd get back to me right away and tell me they're having a little side effect – something that might have made them stop their medicine, or they're feeling bad on this. And I could adjust things before I had time to see them again I really miss it. That was really a help for my practice. It was a huge part of the load of dealing with depression, actually.'

Physicians universally recognized the benefit of care managers. Most physicians with access to care managers regularly used them to contact patients between visits to answer questions, facilitate referrals, monitor adherence and side effects, and assess symptoms. Reasons for not using care managers included not knowing them personally and dissatisfaction with past performance.

'I never found it helpful when some nurse I don't know [provided care management for] somebody. It doesn't give me a picture of anything, so I don't use it. But [I do have access to] that service. If my nurse had time to do it that might be nice, because they know her, and they would tell her what was going on, but she doesn't have time to do it either.'

Also, physicians reported that while they regularly offered to connect patients with care managers, some patients were not interested or were not responsive to care manager contacts. Physicians who had care managers located within their practice (rather than off-site) tended to endorse their benefits more strongly. Physicians who used an electronic medical record system that allowed them to view care management notes including severity assessments appeared to work more closely with and receive more benefits from care managers.

## Discussion

In this study, we described six barriers to depression care. The barriers identified are largely consistent with previous empirical research identifying barriers through physician survey [18-21]. This suggests the same barriers present ten years ago continue to impede primary care physician treatment of depression. One barrier, practice limitations from the fragmented mental health system, was not identified as a major barrier in previous empirical work [18-22], but has been mentioned in editorials and topic reviews [6,34]. The expansion of behavioral health carve-outs and the use of managed care incentives may have intensified this barrier since previous work was conducted [35].

All physicians agreed that education, mental health integration, and care managers were useful in facilitating depression care, suggesting that a high priority should be placed on implementing these interventions because they are most likely to be successful. To realize the full benefit of care management, however, practices need to facilitate coordination, information flow, and relationship building between physicians and care managers, especially for off-site care managers.

Two other interventions, depression screeners and structured assessments, were deemed useful by some physicians but rejected as burdensome by those who believed in their ability to judge depression based on clinical clues and open-ended questions. Educating physicians about the clinical benefits and effectiveness of structured screening and assessment as compared with clinical judgment is needed. Use of opinion leaders and/or academic detailing approaches may also encourage use of these tools. Interventions facilitating consults with psychiatrists received mixed reviews. More physicians perceived this intervention to be more burdensome than helpful; however, few had direct experience with use of consults. Physicians must endorse these interventions before they can be useful in countering barriers and successfully implemented. For practices that have this type of system in place, more education/training about this service or visible leadership adoption may be helpful. For practices considering implementation, initiating discussions to obtain and integrate physician input on implementation may be of value. To facilitate successful implementation, practices may partner with the community and relevant stakeholders on the program design, planning, and implementation [36].

Two systems-related barriers, the fragmented health care system and insurance limitations for mental health, did not appear to be effectively or completely addressed by the interventions. These obstacles were also prominently noted in the Institute of Medicine (IOM) report 'Improving the Quality of Health Care for Mental and Substance-Abuse Conditions: Quality Chasm Series' as well as the President's New Freedom Commission final report [37,38]. Because physicians felt these barriers prevented them from effectively managing patients with depression, future quality improvement interventions should concentrate on strategies that strengthen primary care coordination with mental health specialists and reduce insurance coverage deterrents to both patients' seeking and physicians' providing evidence-based care. Payment and regulatory reform including policies that align incentives to increase collaboration among these providers and mental health parity are also recommended approaches to address these obstacles, and facilitate successful and sustainable implementation of improved depression care [37,39].

Although we found physicians' responses to interventions varied by certain practice characteristics, such as organizational support of the PHQ-9 and the availability of a care manager and other staff support, we did not find variation in the assessments of the interventions stemming from other practice characteristics including size, geographic location, and experience with performance measurement programs. This could be due to limited sample size and requires future study.

Our study contributes to the literature by describing barriers from the physicians' perspective in geographically diverse practices with various exposures to depression interventions. Applying a qualitative methodology in this study allowed us to understand specific mechanisms that influence care and detect previously unidentified barriers to care. Selecting participants purposefully to maximize geographic diversity and represent a variety of practice environments and experiences with quality improvement interventions allowed us to capture physician experiences that have emerged in adapting to different conditions and identify common patterns that cut across practice and geographical variations. Our high response rate given the required time commitment minimized the possibility of selection bias.

Our use of two coders enhanced the reliability of this study by increasing the likelihood that we applied constructs consistently. It also allowed us to generate a deeper understanding of the data. These advantages likely outweigh the additional measure of unreliability introduced by using a second coder.

## Conclusion

Due to the high prevalence and burden of depression, it is critical that barriers are removed in order for primary care physicians to provide evidence-based depression care. Physicians in our study endorse patient education, mental health integration, and the availability of care managers as the more important steps for improving the quality of depression care. These interventions are most likely to be implemented successfully and sustained over time. Systems-related barriers warrant continuous and further attention in discussions surrounding policy, intervention development, and future empirical research.

## Competing interests

JC owns stock in the following for-profit entities: Altria Group Inc, Amer Intl Group Inc (AIG), Colgate Palmolive, CVS Caremark Corp, Hewlett Packard Co, Home Depot Inc, Johnson and Johnson, McDonalds Corp, Medco Health Solutions, Philip Morris Intl Inc, Shaw Group Inc, and Emmis Communications. The remaining authors declare that they have no competing interests.

## Authors' contributions

RH participated in the design of the study, conducted interviews, performed the qualitative analysis and prepared the draft of the manuscript. AC participated in the design of the study, conducted interviews, performed the qualitative analysis, and helped to draft the manuscript. JC participated in the design and coordination of the study, conducted interviews, and helped to draft the manuscript. AZ participated in the design and coordination of the study, conducted interviews, and helped to draft the manuscript. SS conceived of the study, participated in its design and coordination and helped to draft the manuscript. All authors read and approved the final manuscript.
